# The Effectiveness of Hippotherapy to Recover Gross Motor Function in Children with Cerebral Palsy: A Systematic Review and Meta-Analysis

**DOI:** 10.3390/children7090106

**Published:** 2020-08-19

**Authors:** Laura De Guindos-Sanchez, David Lucena-Anton, Jose A. Moral-Munoz, Alejandro Salazar, Ines Carmona-Barrientos

**Affiliations:** 1 Department of Physiotherapy, Upacesur Medical and Functional Rehabilitation Center, 41710 Utrera, Spain; lauraguindos@gmail.com; 2 Department of Physiotherapy, Macrosad Early Childhood Care, 41620 Marchena, Spain; 3 Department of Nursing and Physiotherapy, University of Cadiz, 11009 Cadiz, Spain; ines.carmona@uca.es (I.C.-B.); joseantonio.moral@uca.es (J.A.M.-M.); 4 Institute of Research and Innovation in Biomedical Sciences of the Province of Cadiz (INiBICA), University of Cadiz, 11009 Cadiz, Spain; alejandro.salazar@uca.es; 5 The Observatory of Pain, University of Cadiz, 11009 Cadiz, Spain; 6 Department of Statistics and Operational Research, University of Cadiz, 11009 Cadiz, Spain

**Keywords:** hippotherapy, cerebral palsy, equine-assisted therapy, physical therapy, gross motor function

## Abstract

Cerebral palsy (CP) is a permanent disorder of the posture and movement, which can result in impairments of gross motor function, among others. Hippotherapy (HPT) is an emerging intervention to promote motor recovery in patients with neurological disorders, providing a smooth, precise, rhythmic, and repetitive pattern of movement to the patient. The main objective of this systematic review and meta-analysis of randomized controlled clinical trials was to analyze the effectiveness of HPT interventions on gross motor function in subjects with CP. The following databases were searched in May 2019: PubMed, Scopus, Embase, and Web of Science. The methodological quality of the randomized controlled trials was assessed using the Physiotherapy Evidence Database (PEDro) scale. A total of 10 studies were analyzed in this review, involving 452 participants. Favorable effects were obtained on the gross motor function (Gross Motor Function Measure-66, standardized mean difference (SMD) = 0.81, 95% confidence interval (CI) = 0.47–1.15, Gross Motor Function Measure-88 dimension A SMD = 0.64, 95% CI = 0.30–0.97, dimension B SMD = 0.42, 95% CI = 0.09–0.75, and dimension E SMD = 0.40, 95% CI = 0.06–0.73). The results obtained in the present review show the potential benefit of HPT intervention in improving gross motor function in children with CP.

## 1. Introduction

Cerebral palsy (CP) is the main source of physical disability in children [1]. The prevalence of CP is 2.11/1000 live births since 1985 in high-income developed countries. Children with CP usually present several limitations in terms of postural control, balance, walking, and gross motor function, as well as sensory and perceptual disturbances, spasticity, visual impairment, mental retardation, epilepsy, etc. [2]. These disorders are responsible for inefficient and ineffective movements and activities and it often leads to limitations in carrying out activities of daily living [2]. The neurodevelopmental therapies are usually used in the neurological rehabilitation of children with CP. These therapies are focused on decreasing excessive tone, giving the patient a sense of normal position and movement, and easing normal movement patterns [3].

Hippotherapy (HPT) is an equine-assisted therapy that applies the specific movement of horses in the rehabilitation of patients with neurological disorders [4], improving the neurological functions and sensory processes [5,6]. The research in HPT has increased in recent years as a complementary therapy to traditional treatments [6]. HPT is based on two main action mechanisms: (i) the transmission of the warmth and (ii) the transmission of three-dimensional movements with rhythmic impulses from the horse to the patient’s body. The pelvis of the patient is moved in a repetitive, rhythmic, and soft pattern, which is similar to the movement carried out during human gait. This three-dimensional movement stimulates balance reactions, improves postural balance and the trunk straightening [4]. This therapy provides movements in all the movement planes, coming from the alternating elevation of the horse’s back that originate anteversion/retroversion, elevation/decrease, and lateral movement with rotation [5]. In addition, HPT provides sensory input and induces greater postural control and motor responses [6]. Several favorable physical effects of HPT were found in muscle coordination, muscle tone, balance, posture, strength, endurance, and flexibility, improving gait and patterns of abnormal movement. In addition, it also showed positive improvements at the social, cognitive, and psychological levels [6]. Furthermore, several recent reviews suggested that HPT could be effective for the neurological rehabilitation of subjects with CP: Novak et al. stated that HPT was a successful allied health therapy to improve muscle symmetry in subjects with CP [7]; Mendizábal Alonso [8] suggested that HPT was effective to improve postural alignment in subjects with CP; Martin-Valero et al. [9] also reported benefits in the performance of the activities of daily living and quality of life; and Zadnikar and Kastrin [10] also obtained favorable results on postural balance in subjects with CP.

Regarding the motor function of children with CP, the main aim of therapeutic interventions is to increase the performance of the gross motor skills that are key components of the functional mobility [11]. To the best of our knowledge, only a systematic review carried out in 2012 by Whalen and Case-Smith [12] suggested that HPT could produce benefits on gross motor function in subjects with CP. Therefore, the current evidence through meta-analysis analyzing the use of HPT to recover gross motor function in patients with CP is limited. Consequently, the aim of this systematic review and meta-analysis is to evaluate the effectiveness of HPT for improving gross motor function in children with CP.

## 2. Materials and Methods

### 2.1. Search Strategy

The present review was carried out following the preferred reporting items for systematic reviews and meta-analyses (PRISMA) [13] recommendations for systematic reviews. The literature search was carried out using the databases: PubMed, Web of Science (WoS), Scopus, and Embase. The search covered up to May 2019, without a limit in the starting date. It was performed by combining the following keywords: “hippotherapy” and “cerebral palsy”. No filters were applied in relation to the publication dates or language, but the results were filtered to obtain only studies that corresponded to randomized clinical trials (RCTs).

### 2.2. Selection Criteria

The articles included in this review met the following inclusion criteria based on the PICOS model: (P) population: subjects diagnosed with CP; (I) intervention: HPT; (C) comparison: with conventional physical therapy intervention or placebo; (O) outcomes: gross motor function; and (S) study design: RCTs. The exclusion criteria were: (I) studies that involved healthy participants; (II) more than one intervention compared in the study; and (III) an intervention performed using HPT simulators. In addition, we excluded articles in which the intervention was based on therapeutic riding because the instructors may not always be medical professionals using an interdisciplinary team approach [10].

### 2.3. Study Selection Process and Data Extraction

First, a literature search was conducted in the scientific databases by combining keywords. Afterwards, we identified and excluded the duplicated articles. After this first selection, the titles and abstracts of the articles found were reviewed. Next, a second exclusion process was made of those studies that did not fulfill the inclusion criteria. These articles obtained after this last selection were evaluated in depth to fulfill the specific inclusion criteria. Finally, the studies that form part of this review were included. Two reviewers (L.D.-G.S. and D.L.A.) independently selected, reviewed, and extracted data form the studies. An additional reviewer (I.C.B.) participated in the consensus of the decisions. We extracted the following information from each study: author, year of publication, number of participants from both groups, average age, gender, levels of the gross motor function classification system (GMFCS), type of CP, intervention carried out, frequency, duration, outcomes, measuring instruments, and results.

### 2.4. Assessment of the Methodological Quality of the Studies

The PEDro [14] was used to assess the methodological quality of the studies. This scale comprises different items in terms of the following domains: performance, selection, information, detection, and attribution bases. A higher score shows a higher methodological quality. A study with a PEDro score of 6 or higher is considered as evidence level 1 (6–8 is good; 9–10 is excellent), and a study with a score of 5 or less is considered as evidence level 2 (4–5 is acceptable; <4 is poor) [15].

## 3. Results

**Figure 1:**
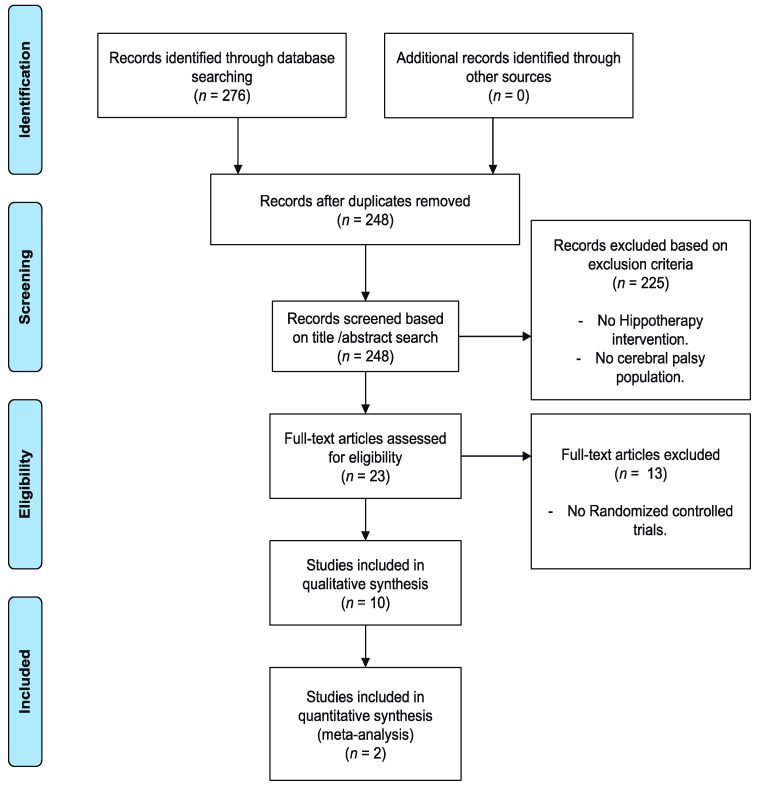
Flow diagram of included articles.

Once the database searches were completed, using the different keywords, a total of 276 documents were obtained, as shown in Figure 1. Finally, 10 studies met the inclusion criteria for review.

### 3.1. Methodological Quality of the Studies

Table 1 shows the PEDro scores achieved by the articles reviewed in this study. Three of ten articles were considered to have a high methodological quality: McGibbon et al. [16]; Kwon et al. [17], and Lucena-Antón et al. [5]. Matusiak-Wieczorek et al. [18] achieved the lowest score. The overall methodological quality was acceptable (average total score = 5.1, range 3–7).

### 3.2. Main Characteristics of the Studies Included in the Systematic Review

Regarding the age of the participants, the highest average age among the control groups was found in the study by McGibbon et al. [16] (8.8 years), while among the intervention groups, it was found in the study by Lucena-Antón et al. [5] (9.6 years). The lowest average age in both groups was presented in the study by Kwon et al. [17] (5.9 and 5.7 years, respectively). In terms of the sample size, the study by Kwon et al. [17] achieved the highest sample size with a total of 91 participants. The overall sample size ranged from 15 to 73 subjects. Table 2 shows the main clinical and demographic characteristics of the participants.

Concerning the different effects analyzed in the different studies, three studies [17,22,23] analyzed the effects of HPT interventions on gross motor function, four studies [17,18,20,23] analyzed the effects on balance, two studies [5,23] analyzed the spasticity, and two studies [16,19] analyzed the muscle activity through electromyography. The main intervention characteristics of the studies included in the systematic review are shown in Table 3.

### 3.3. Meta-Analysis of the Study Groups

The groups were created according to the measuring instrument used to assess the gross motor function. Accordingly, seven groups were set up: (i) GMFM-66 total scores; (ii) GMFM-88 total scores; and (iii–vii) GMFM-88 dimensions A–E.

The gross motor function measure (GMFM) is commonly used in neurological rehabilitation to assess the gross motor function in subjects with CP. The GMFM-66 scale is an updated version of GMFM-88. It includes 66 of the original 88 items providing more information to encourage the goal setting process [11]. Both scales include different items that assess how much of an activity can be carried out rather than the quality of performing the activities [25]. Both versions have been validated to evaluate changes in children with CP. A higher score is an indicator of better gross motor function [26].

**Figure 2:**
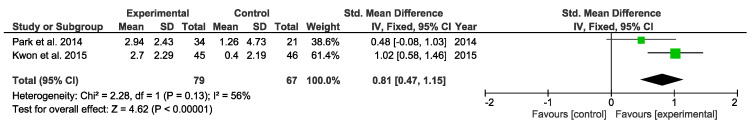
Forest plot for gross motor function measured by the GMFM-66 scale.

Two studies analyzed the differences in gross motor function using the GMFM-66. The overall result of this study group was favorable (Figure 2).

**Figure 3:**
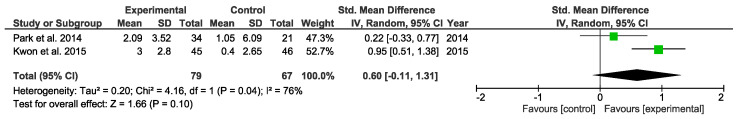
Forest plot for gross motor function measured by the GMFM-88 total scores.

Regarding the GMFM-88 scale, it is divided into five dimensions (A: lying and rolling, B: sitting, C: crawling and kneeling, D: standing, and E: walking, running, and jumping). The total score ranges from 0 to 100. For the GMFM-88 total score, the overall result of the meta-analysis was not conclusive (Figure 3).

**Figure 4:**
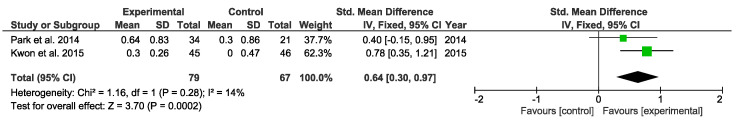
Forest plot for GMFM-88 Dimension A.

**Figure 5:**
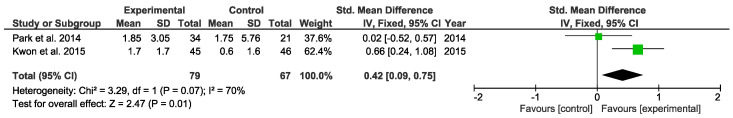
Forest plot for GMFM-88 Dimension B.

**Figure 6:**
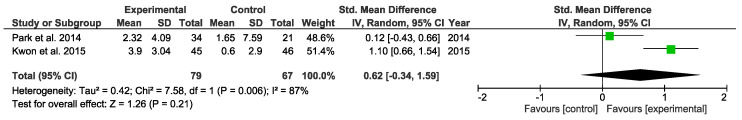
Forest plot for GMFM-88 Dimension C.

**Figure 7:**
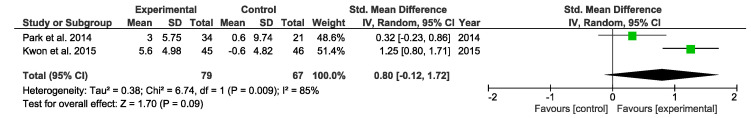
Forest plot for GMFM-88 Dimension D.

**Figure 8:**
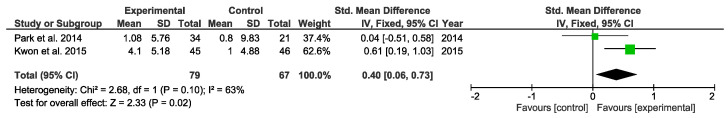
Forest plot for GMFM-88 Dimension E.

Regarding the different dimensions included in the GMFM-88 scale, the overall result of the meta-analysis was favorable in GMFM-88 dimensions A, B, and E, while the overall result of the meta-analysis was inconclusive for GMFM-88 dimensions C and D. The results are shown in Figure 4, Figure 5, Figure 6, Figure 7 and Figure 8.

## 4. Discussion

The objective of this systematic review and meta-analysis of RCTs was to analyze the effectiveness of HPT interventions on improving gross motor function in subjects with CP. A total of ten RCTs were analyzed in the systematic review, involving 452 participants. In view of our results, we could conclude that HPT could be an effective intervention to improve gross motor function in children with CP.

From a clinical perspective, the findings obtained in the present review suggest that HPT stands for an emerging intervention in neurological rehabilitation, which could be used in addition to neurodevelopmental based methods. The findings on the GMFM-66 scale and GMFM-88 dimensions A, B, and E showed that HPT interventions had significant improvements on gross motor function and, more specifically, on the ability to perform lying and rolling, sitting, and walking. We can hypothesize that the rhythmic and symmetrical movement of the horse could stimulate the proprioception and balance reactions. Furthermore, according to Casady and Nichols-Larsen [27], HPT could stimulate the motor learning and subjects could be able to transfer the movement patterns learned from HPT to other usual environments. According to Bertoti [28], and considering that three of four studies [17,18,20,23] did obtain significant effects on balance and two studies [5,23] reported significant effects on the spasticity of hip adductors, we can suggest that the positive effects obtained on balance and muscle spasticity contributed to improvements in the functional outcomes and, thus, to the significant results obtained in the GMFM-66 and GMFM-88 dimensions.

Regarding the intervention characteristics, most studies included more than 30 participants, a high number considering the difficulty to recruit patients with CP. All intervention groups received HPT in addition to physical therapy, and all of them carried out their HPT interventions through a walking pace, except for Alemdaroğlu et al. [23] and Deutz et al. [24] that did not specify it. Most studies used protocols with 8–12 weeks as the total duration and two times/week as the frequency. The session duration used in the studies was around 30 min, with unusual interventions of more than 45 min. The effects found were similar and several authors suggested that longer durations could cause fatigue in children, which was not positive for achieving the intended improvements. Therefore, we can suggest that HPT interventions based on 8–12 week programs with sessions of 30–45 min two times a week could be proper for children with CP to recover motor function.

Concerning the methodological quality of the studies included in the present review, the main limitation was found in the application of double-blind. Blinding of the participants and therapists was not possible in most studies due to the unconcealable nature of the intervention. In addition, the concealed allocation was only possible in two studies [16,19] and the assessor blinding was carried out by four studies [5,16,17,24]. Nevertheless, the overall quality of the studies was acceptable.

Our results matched with the findings of the systematic review conducted by Whalen and Case-Smith [12] in 2012, in which they stated that HPT could produce benefits on gross motor function in children with CP, but the authors highlighted that the evidence was limited. Other findings were found in different pathologies, such as Down syndrome and autism disorder. De Miguel Rubio et al. [29] suggested that HPT could not be effective to improve gross motor function in subjects with Down syndrome, and Srinivasan et al. [30] analyzed the effects of HPT interventions in subjects with autism disorder, obtaining positive effects on motor skills.

The present systematic review presented some limitations. Potential useful articles that were indexed in other scientific databases could not be included. In addition, the lack of long-term follow-up and the heterogeneity of the protocols suggests the need to unify the HPT intervention programs, specifically, in subjects with CP. Moreover, despite assessing the same outcomes between the different studies included in the review, the statistical comparison was not always possible due to studies used different scales and measuring instruments to assess the clinical differences. Thus, only two studies were included in the meta-analysis. Therefore, the results obtained should be taken with caution since a limited number of studies was analyzed.

## 5. Conclusions

In conclusion, we could state that HPT interventions were effective to improve gross motor function in subjects with CP. Specifically, favorable results were obtained in the GMFM-66 total scores and GMFM-88 dimensions A, B, and E. Furthermore, positive effects have been showed on balance recovery and muscle spasticity reduction.

Despite the different HPT protocols used, evidence shows that 30–45 min sessions, twice weekly for 8–12 weeks, could produce significant effects on gross motor function in children with CP.

This study can be helpful in neurological rehabilitation of children with CP using HPT interventions, as well as by providing key factors to determine which specific factors of the HPT protocols have a greater weight to achieve the desired effects in future interventions. Nevertheless, it will be necessary to carry out more randomized controlled trials with larger sample sizes and specified protocols.

## Figures and Tables

**Table 1:** Analysis of the methodological quality of the studies (PEDro scores).

Study	1	2	3	4	5	6	7	8	9	10	11	Total
Benda et al., 2003 [19]	-	Yes	Yes	No	No	No	No	Yes	No	Yes	Yes	5
McGibbon et al., 2009 [16]	-	Yes	Yes	Yes	No	No	Yes	Yes	No	Yes	Yes	7
Kang et al., 2012 [20]	-	Yes	No	Yes	No	No	No	Yes	No	Yes	Yes	5
El-Meniawy and Thabet 2012 [21]	-	Yes	No	Yes	No	No	No	No	No	Yes	Yes	4
Park et al., 2014 [22]	-	Yes	No	Yes	No	No	No	Yes	No	Yes	Yes	5
Kwon et al., 2015 [17]	-	Yes	No	Yes	Yes	No	Yes	Yes	No	Yes	Yes	7
Matusiak-Wieczorek et al., 2016 [18]	-	Yes	No	No	No	No	No	No	No	Yes	Yes	3
Alemdaroglu et al., 2016 [23]	-	Yes	No	Yes	No	No	No	No	No	Yes	Yes	4
Deutz et al., 2017 [24]	-	Yes	No	Yes	No	No	Yes	No	No	Yes	No	4
Lucena-Antón et al., 2018 [5]	-	Yes	No	Yes	No	No	Yes	Yes	Yes	Yes	Yes	7

Range: 0–10. Item 1 is not included in the total score. Item 1: Eligibility criteria; Item 2: Random allocation; Item 3: Concealed allocation; Item 4: Baseline similarity; Item 5: Subject blinding; Item 6: Therapist blinding; Item 7: Assessor blinding; Item 8: >85% follow up; Item 9: Intention-to-treat analysis; Item 10: Between-group statistical comparison; Item 11: Point and variability measures.

**Table 2:** Main clinical and demographic characteristics of the participants.

Study	Participants (*n*)	Age (Years) ± SD	Female/Male	GMFCS Levels	Type: Diplegia/Hemiplegia (*n*)	Diagnosis
Benda et al., 2003 [19]	IG: (*n* = 7)	4–12	ND	ND	ND	Spastic (*n* = 15)
	CG: (*n* = 8)					
	*N* = 15					
McGibbon et al., 2009 [16]	Phase 1:	IG: 8.5	IG: 9/16	I: (*n* = 27)	IG: 12/4	Spastic (*n* = 38)
	IG: (*n* = 25)			II: (*n* = 9)		
	CG: (*n* = 19)				CG: 13/3	
	*N* = 44	CG: 8.8	CG: 11/11	III: (*n* = 5)		Mixed (*n* = 6)
	Phase 2:				Quadriplegia: 9	
	IG: (*n* = 6)			IV: (*n* = 6)		
Kang et al., 2012 [20]	IG1:(*n* = 14)	IG1: 8.2 ± 1.1	IG1: 7/7	ND	IG1: 5/9	ND
	IG2: (*n* = 15)	IG2: 8.2 ± 1.2	IG2: 7/8		IG2: 5/10	
	CG: (*n* = 15)	CG: 7.8 ± 1.5	CG: 7/7		CG: 5/9	
	*N* = 44					
El-Meniawy and Thabet 2012 [21]	IG: (*n* = 15)	7.02 ± 0.5	ND	ND	ND	Spastic (*n* = 30)
	CG: (*n* = 15)					
	*N* = 30					
Park et al., 2014 [22]	IG: (*n* = 34)	IG: 6.68 ± 2.6	IG: 19/15	I: (*n* = 14)	IG: 32/2	Spastic (*n* = 55)
	CG: (*n* = 21)	CG: 7.76 ± 3.7	CG: 11/10	II: (*n* = 15)		
				III: (*n* = 11)	CG: 19/2	
				IV: (*n* = 15)		
	*N* = 55					
Kwon et al., 2015 [17]	IG: (*n* = 45)	IG: 5.7 ± 1.9	IG: 25/20	I: (*n* = 24)	IG: 41/4	Spastic (*n* = 84)
	CG: (*n* = 46)			II: (*n* = 24)		
	*N* = 91	CG: 5.9 ± 1.8	CG: 17/29	III: (*n* = 23)	CG: 40/6	Dyskinetic (*n* = 4)
				IV: (*n* = 20)		Ataxic (*n* = 3)
Matusiak-Wieczorek et al., 2016 [18]	IG: (*n* = 19)	IG: 8.42 ± 2.2	IG: 9/10	I: (*n* = 23)	IG: 6/13	Spastic (*n* = 39)
	CG: (*n* = 20)	CG: 8.3 ± 2.6	CG: 9/11	II: (*n* = 16)	CG: 5/15	
	*N* = 39					
Alemdaroğlu et al., 2016 [23]	IG: (*n* = 9)	7.5 ± 1.7	7/9	IG: I–IV	ND	Spastic (*n* = 16)
	CG: (*n* = 7)			CG: I–V		
	*N* = 16					
Deutz et al., 2017 [24]	IG: (*n* = 35)	IG: 9.29 ± 3.7	IG: 12/23	II: (*n* = 27)	IG: 35/0	Spastic (*n* = 73)
	CG: (*n* = 38)	CG: 8.87 ± 2.9	CG: 17/21	III: (*n* = 17)	CG: 38/0	
	*N* = 73			IV: (*n* = 29)		
Lucena-Antón et al., 2018 [5]	IG: (*n* = 22)	IG: 9.5 ± 2.7	IG: 9/13	IV–V	ND	Spastic (*n* = 44)
	CG: (*n* = 22)	CG: 8.2 ± 2.4	CG: 7/15			
	*N* = 44					

CG: control group; GMFCS: Gross Motor Function Classification System; IG: Intervention group; ND: Not described.

**Table 3:** Summary of interventions carried out by the different studies included in the systematic review.

Study	Participants	Intervention	Frequency	Session Duration	Total Duration	Outcomes	Measuring Instruments	Results
Benda et al., 2003 [19]	IG: (*n* = 7)	IG: HPT	One session	8 min	One session	Muscle activity in the paravertebral, hip abductors/adductors when sitting, standing, and walking	EMG	IG got better results than CG. Mean change improvements: IG = 64.6% (SD = 28.3) vs. CG = −12.8% (SD = 88.8); (*p* = 0.051)
	CG: (*n* = 8)	CG: Exercises on a barrel						
McGibbon et al., 2009 [16]	Phase 1:	IG: HPT	Phase 1: One session	Phase 1: 10 min	Phase 1: One session	Hip adductors muscle activity	SEMG	Phase 1: The IG significantly improved the muscle asymmetry of hip adductors (*p* < 001; *d* = 1.32)
	IG: (*n* = 25)							
	CG: (*n* = 19)							
	Phase 2:	CG: Exercises on a barrel	Phase 2: Once a week	Phase 2: 40 min	Phase 2: 36 weeks			Phase 2: After 12 weeks, 4 of 6 children improved the muscle symmetry of hip adductors
	IG: (*n* = 6)							
Kang et al., 2012 [20]	IG1:(*n* = 15)	IG1: HPT	Once a week	30 min	8 weeks	Sitting balance	Force plate	The results showed that pathway and velocity significantly decreased in the HPT group (*p* < 0.05) compared to the PT and CON groups
	IG2: (*n* = 15)	IG2: PT						
	CG: (*n* = 15)	CG: Non treatment						
El-Meniawy and Thabet 2012 [21]	IG: (n = 15)	IG: HPT	IG: Once a week	IG: 30 min	12 weeks	Back geometry parameters: lateral deviation, trunk imbalance, pelvic tilt, rotation	Formetric instrument system	The results showed improvements in favor of the IG in all the outcomes (*p* < 0.05)
	CG: (n = 15)	CG: Exercise	CG: 3 times/week	CG: 1 h				
Park et al., 2014 [22]	IG: (*n* = 34)	IG: HPT	2 times/week	45 min	8 weeks	Gross motor function	GMFM-66	Significant results were obtained in IG after the intervention compared to the CG: GMFM-66 (all dimensions); GMFM-88 (B and C dimensions); and 3 domains of the PEDI-FSS: (*p* < 0.05)
	CG: (*n* = 21)	CG: Non treatment				Functional performance	GMFM-88	
							PEDI-FSS	
Kwon et al., 2015 [17]	IG: (*n* = 45)	IG: HPT	2 times/week	30 min	8 weeks	Gross motor function.	GMFM-66	Significant results were found between groups (*p* < 0.05): GMFM- 66, GMFM-88 (total score and dimensions B, C, D, and E). Moreover, significant results were found in balance (*p* < 0.05)
	CG: (*n* = 46)	CG: Aerobic exercise				Balance	GMFM-88 PBS	
Matusiak-Wieczorek et al., 2016 [18]	IG: (*n* = 19)	IG: HPT	Once a week	30 min	12 weeks	Body balance in sitting position	SAS	Significant results were obtained in IG for arm function and control of trunk position: (*p* = 0.018)
	CG: (*n* = 20)	CG: NI						
Alemdaroğlu et al., 2016 [23]	IG: (*n* = 9)	IG: HPT	IG: 2 times/week	30 min	5 weeks	Gross motor function, hip adductors spasticity, balance, hip abduction angle, knee distance	GMFMCS	Significant improvements were observed between groups in spasticity (*p* = 0.016). Not significant results were found in other outcomes
							MAS	
	CG: (*n* = 7)	CG: PT	CG: 5 times/week				MFRT	
							Goniometer	
Deutz et al., 2017 [24]	IG: (*n* = 35)	IG: HPT	1–2 times/week	ND	16–20 weeks	Gross motor function and quality of life	GMFM-66 KIDSCREEN-27 questionnaire	Improvements were observed in GMFM-66 dimension E for IG (*p* = 0.02) compared to CG. Not significant results were found in quality of life
	CG: (*n* = 38)	CG: PT					CHQ	
Lucena-Antón et al., 2018 [5]	IG: (*n* = 22)	IG: HPT	IG: Once a week	45 min	12 weeks	Hip adductors spasticity	MAS	Significant results were obtained between groups for IG in spasticity (*p* = 0.04 for left adductors and *p* = 0.047 for right adductors)
	CG: (*n* = 22)	CG: PT	CG: 2 times/week					

CG: control group; CHQ: Child Health Questionnaire; EMG: Electromyography; GMFM: Gross Motor Function Measure; HPT: Hippotherapy; IG: Intervention group; MAS: Modified Ashworth Scale; MFRT Modified Functional Reach Test; Min: Minutes; ND: not described; PBS Pediatric Balance Scale; PEDI-FSS: Pediatric Evaluation of Disability Inventory-Functional Skills Scale; PT: Physical therapy. SAS Sitting Assessment Scale; PDM Multifunction Force Measure Plate; SD: Standard deviation; SEMG: Surface Electromyography.
